# Letter to the Editor: Effects of Minimally Processed Red Meat within a Plant-Forward Diet on Biomarkers of Physical and Cognitive Aging—A Randomized Controlled Crossover Feeding Trial

**DOI:** 10.1016/j.cdnut.2026.109537

**Published:** 2026-09-22

**Authors:** Miguel López-Moreno

**Affiliations:** 1 Diet, Planetary Health and Performance, Faculty of Health Sciences, Universidad Francisco de Vitoria, Pozuelo, Spain; 2 Institute of Health and Sport Sciences, Faculty of Health Sciences, Universidad Francisco de Vitoria, Madrid, Spain

Dear Editor:

I read with great interest the randomized crossover feeding trial by Vaezi et al. [1] evaluating minimally processed pork compared with pulse-based meals within a plant-forward dietary pattern. The study represents a considerable methodological achievement, and I congratulate the authors for conducting such a demanding controlled-feeding intervention. Beyond its specific findings, however, I believe the trial raises broader methodological questions regarding the design and interpretation of food-based randomized controlled trials (RCTs).

According to the accompanying methodology paper, the interventions were specifically designed to minimize differences in nutrient composition by matching energy, macronutrients, fiber, and several micronutrients through the incorporation of additional foods and ingredients into both intervention arms [2]. Thus, soluble fiber and nutritional yeast were incorporated into the meat intervention, whereas eggs, dairy products, pea protein, and commercially available plant-based products were incorporated into the pulse intervention to improve nutritional comparability. The interventions were further designed to closely match dietary fiber and other nutritional characteristics.

Although nutrient matching undoubtedly strengthens internal validity by reducing differences in nutrient intake, I believe it also raises an important question regarding causal inference. Fiber, SFAs, amino acid composition, and other characteristics of whole foods are not merely potential confounders of meat and legume consumption. Rather, they are intrinsic properties of the foods themselves and may constitute part of the causal pathway through which dietary substitutions exert their physiological effects. Matching these characteristics therefore differs conceptually from controlling traditional confounding.

For this reason, this design also raises the possibility of what might be considered a form of design-induced overmatching, whereby characteristics intrinsic to the intervention itself are intentionally harmonized across comparison groups, potentially attenuating the biological contrast the trial aims to evaluate. In such circumstances, the causal question gradually changes. Rather than evaluating the physiological consequences of replacing minimally processed meat with legumes, the intervention increasingly evaluates the consequences of replacing meat with legumes after many of their intrinsic nutritional differences have already been minimized.

This issue appears particularly relevant because the accompanying methodology paper explicitly adopts a whole-diet and food-matrix perspective, recognizing that physiological responses emerge from interactions among foods and dietary components rather than isolated nutrients [3]. Yet, extensive nutrient matching may inadvertently reduce precisely those food-matrix characteristics that distinguish the interventions. From a causal inference perspective, both questions are scientifically legitimate, but they represent different estimands and therefore require different interpretations [4,5]. As recently discussed in conceptual frameworks on nutritional causal inference, valid interpretation depends on maintaining alignment among the causal question, intervention definition, comparator, analytical strategy, and interpretation of findings [6].

A second aspect that merits clarification concerns the interpretation of study outcomes. According to the published methodology, sample size calculations were based primarily on homocysteine because it required the largest sample size among the prespecified primary outcomes. Consequently, this represents the endpoint for which the study was prospectively powered, whereas several additional outcomes were explicitly considered exploratory. Nevertheless, despite homocysteine showing one of the clearest between-group differences favoring the pulse intervention, the discussion and conclusions devote comparatively greater attention to exploratory findings. Greater alignment between the prespecified statistical hierarchy and the interpretation of results would improve transparency and facilitate a more balanced interpretation of the evidence.

Finally, this discussion extends beyond the present trial. Food-based RCTs inevitably face the challenge of balancing internal validity with preservation of meaningful dietary contrasts. Improving one objective should not inadvertently compromise the others. Future trials may therefore benefit from explicitly distinguishing between matching genuine confounding characteristics and matching characteristics that constitute intrinsic components of the dietary exposure itself. Doing so may help preserve the causal contrast under investigation while maintaining the biological realism necessary for clinically meaningful dietary recommendations.

## Author contributions

The sole author was responsible for all aspects of this manuscript.

## Data availability

Data sharing does not apply to this article as no datasets were generated or analyzed during the current study.

## Declaration of Generative AI and AI-assisted Technologies in the Writing Process

Generative AI was used exclusively to improve grammar, spelling, and language clarity during manuscript preparation. No AI tools were used to generate scientific content, interpret results, perform analyses, or draw conclusions. The author reviewed and approved all revisions and assumes full responsibility for the final content of the manuscript.

## Funding

The author reported no funding received for this study.

## Conflict of interest

ML-M reports having previously received remuneration from Danone and Foods For Tomorrow for advisory board participation and consulting activities. These activities were unrelated to the submitted work and are not ongoing. At present, he has no active financial relationships with these entities.
